# Neural representations in mPFC and insula encode individual differences in estimating others’ preferences

**DOI:** 10.1093/scan/nsaf051

**Published:** 2025-05-14

**Authors:** Hyeran Kang, Kun Il Kim, Jinhee Kim, Hackjin Kim

**Affiliations:** Department of Psychology, University of Michigan, Ann Arbor, MI 48109, USA; Laboratory of Social and Decision Neuroscience, School of Psychology, Korea University, Seoul, 02841, Republic of Korea; Laboratory of Social and Decision Neuroscience, School of Psychology, Korea University, Seoul, 02841, Republic of Korea; Laboratory of Social and Decision Neuroscience, School of Psychology, Korea University, Seoul, 02841, Republic of Korea; Laboratory of Social and Decision Neuroscience, School of Psychology, Korea University, Seoul, 02841, Republic of Korea

**Keywords:** inter-subject representational similarity analysis, anterior cingulate cortex, theory of mind, interoception, face

## Abstract

In human society, successful social interactions often hinge upon the ability to accurately estimate other’s perspectives, a skill that necessitates integrating contextual cues. This study investigates the neural mechanism involved in this capacity through a preference estimation task. In this task, participants were presented with the target’s face and asked to predict their preference for a given item. Preference estimation accuracy was assessed by calculating the percentage of correct guesses, where participants’ responses matched the target’s preferences on a 4-point Likert scale. Our research demonstrates that, based on inter-subject representational similarity analysis (IS-RSA), the multi-voxel patterns in the medial prefrontal cortex (mPFC) and the anterior insula (AI) predict individual differences in preference estimation accuracy. Specifically, the varying behavioural tendencies among participants in inferring others’ preferences were mirrored in the multivariate neural representations within these regions, both of which are known for their involvement in individual differences in interoception and context-dependent interpretation of ambiguous facial emotion. These findings suggest that mPFC and AI play pivotal roles in accurately estimating others’ preferences based on minimal information and provide insights that transcend the limitations of traditional univariate approaches by employing multivariate pattern analysis.

## Introduction

In daily life, people often form impressions of others and make judgments based on highly limited information, which is referred to as ‘thin-slicing’ in psychology (Ambady and Rosenthal 1992). Such an ability demonstrates how people use fast and automatic processes to make decisions, such as interpreting nonverbal cues like facial expressions, gestures, and tone of voice during interactions. For example, reading a text about a person’s major life events can predict their personality (Pennebaker and King 1999), and simply observing someone’s room can accurately judge their personality (Gosling et al. 2002). Moral judgments can be made from brief visual scenes (De Freitas and Hafri 2024), and after watching a 60-second video of a social interaction, one can predict the other person’s socioeconomic status (Kraus and Keltner 2009).

Among others, facial cues are particularly integral to social inference, serving as a rich source of insight into an individual’s traits (Zebrowitz et al. 2002, Frith and Frith 2012). Previous studies have shown that, in less than 100ms, one can infer how trustworthy someone is (Willis and Todorov 2006), predict a company’s financial performance by looking at the CEO’s face (Rule and Ambady 2008b), accurately assess someone’s sexual orientation (Rule and Ambady 2008a), and predict the outcomes of political candidates in elections (Todorov et al. 2005).

Numerous psychological literatures highlight the significance of intuitive social inferences, in which individuals are tasked with inferring affective states or psychological traits of targets. These tasks encompass estimating transient emotional states as interpreted through facial expressions (Frith and Frith 2012), understanding intentions of approach and avoidance (Jones and Kramer 2021), and discerning psychological traits such as trustworthiness (Van’t Wout and Sanfey 2008, Chwe and Freeman 2023), cooperativeness (Tognetti et al. 2013), intelligence (Zebrowitz et al. 2002), and preferences (North et al. 2010, 2012, Kang et al. 2013, Eggleston et al. 2015, Pollmann and Scheibehenne 2015, Vijayakumar et al. 2021). These intuitive social inferences, based on facial information, are integral components of understanding others, including empathy and the theory of mind (Quesque and Rossetti 2020), yet the exact neural mechanisms behind these instinctual inferences require further exploration.

What factors determine the accuracy of predicting others’ traits based on limited information, such as a face? Research conducted to answer this question so far suggests that higher emotional recognition ability (Jaksic and Schlegel 2020) and intuitive over analytical thinking (Albrechtsen et al. 2009) are associated with higher accuracy in thin-slicing. Compared to the vast psychological research on thin-slicing, studies on the neurological mechanisms related to this phenomenon are relatively scarce, especially regarding the biological factors that differentiate individuals with high and low accuracy in thin-slicing. Research that identifies the neurological characteristics underlying individual differences in thin-slicing accuracy is expected to provide important insights into distinguishing innate from acquired factors in this phenomenon.

Our laboratory has previously reported on the significant role of the dorsomedial prefrontal cortex (dmPFC) communicating with the temporoparietal junction (TPJ) in predicting others’ preferences based on facial features and the associated individual differences in accuracy (Kang et al. 2013, Park et al. 2018). Prior research in social cognition suggests that the dmPFC, in concert with the TPJ, forms the core of the mentalizing network (Schnell et al. 2011, Dvash and Shamay-Tsoory 2014). Meta-analyses indicate that dmPFC activities are engaged when observing others receiving painful stimuli (Lamm et al. 2011), estimating others’ thoughts through perspective-taking, and inferring their emotional states (Schurz et al. 2014, 2021), emphasizing its role in abstract representation based on external information for generating appropriate affective and cognitive behaviours (Kim 2020). Beyond mentalizing studies, the dmPFC is involved in generating new actions based on prediction errors and learning abstract rules (Seo et al. 2014) and representing other-regarding values for prosocial decisions (Sul et al. 2015). Increased dmPFC activity during the observation of social scenarios correlates with a higher frequency of social interactions (Powers et al. 2016). Similarly, dmPFC activities are instrumental in predicting others’ behaviour (Amodio and Frith 2006) and showed a positive correlation with the accuracy of preference estimation based on facial appearance (Kang et al. 2013).

Multivariate pattern analysis (MVPA) is a statistical technique used to analyse brain imaging data. Unlike traditional univariate analysis, which examines activity of one voxel at a time, MVPA analyzes patterns of activity across multiple voxels simultaneously, making it more sensitive to detecting subtle differences between psychological states (Norman et al. 2006). Supporting evidence shows that multivariate analyses reveal unique activity patterns in brain regions that are commonly activated across conditions in univariate analyses (Woo et al. 2014, Krishnan et al. 2016, Wake and Izuma 2017, Kim and Kim 2021). Considering the differences between these two methods, it is necessary to determine whether the role of the dmPFC, linked to individual differences in the accuracy of predicting others’ preferences, remains valid in both univariate and multivariate analyses, or if there are specific differences between the two methods related to these individual differences.

In this study, we delve into the neural mechanisms underlying individual variations in preference estimation accuracy. First, we aimed to replicate the findings regarding the role of the dmPFC in accurately evaluating other’s preferences, as previously reported by Kang et al. (2013). Secondly, we opted to capitalize on recent advances in MVPA techniques, renowned for their capability to map hidden neural representations of psychological traits and motivations that could not be captured by conventional univariate approaches (Kragel et al. 2021, Contreras-Huerta et al. 2023). To achieve this, we employed a preference estimation task, where participants were shown an image of a person followed by an image of a food or movie poster (Kang et al. 2013) and asked to infer the preference of the person to the image solely based on their facial appearances. Our study leveraged inter-subject representational similarity analysis (van Baar et al. 2019) to identify the specific brain regions where activity patterns are correlated with individual variability in the accuracy of preference estimation.

## Materials and methods

### Participants

A total of 39 healthy female participants, aged between 22 and 44 (all females; mean age = 30.82), were recruited for this study. The decision to recruit only female participants was based on prior research (Carney et al. 2007), which suggests that female participants tend to make more accurate thin-slice judgments compared to male participants. By selecting a single gender cohort, we sought to minimize potential gender effects. All participants were right-handed, had no visual impairments, and reported no history of psychiatric or neurological conditions. However, due to artefacts in neuroimaging data, one participant’s data was excluded. Thus, final analyses were conducted on 38 participants (age range: 22–44, mean age = 30.56). To determine whether our study had sufficient statistical power, we conducted a Bayesian power analysis. Unlike traditional frequentist power analysis, which assumes a fixed effect size, Bayesian approaches incorporate empirical uncertainty to provide a more robust and realistic assessment of power (Du and Wang 2016, Kruschke and Liddell 2018). Based on our observed effect size (*r* = .566) and a final sample size (N = 38), the probability that the true effect size exceeds *r* = .34 is 95.27%. This threshold of *r* = .34 was derived from a transformation of the effect size reported in Kang et al. (2013). These results suggest that our study was well-powered to detect meaningful effects within the expected range, thereby confirming the robustness of the hypothesized relationship. All participants provided informed consent, in accordance with the guidelines set by the Institutional Review Board of Korea University. These participants performed a facial expression recognition task prior to this task, and these data have already been published elsewhere (Kim et al. 2022).

### Procedure

Upon arrival, participants’ eligibility for MRI scanning was verified. Before scanning, participants were given instructions and completed a couple of practice trials to familiarize themselves with the experimental paradigm. The primary task was the preference estimation paradigm adapted from Kang et al. (2013), designed to investigate neural mechanisms associated with inferring preferences (e.g., for movie posters or food items) based on facial photographs of targets. The photos of targets were collected from an independent group of eight individuals (four males, four females; all Korean) who had provided their preferences for five distinct food items and five distinct movie stimuli.

Following the methodology established by Kang et al., we selected food and movie posters as stimuli to best allow participants to estimate others’ preferences accurately. In their study, various item categories were evaluated, including movies, books, bags, shoes, and foods, to determine which categories would best facilitate accurate preference estimation. Items were selected based on preference ratings, focusing on those with intermediate levels of preference and high variability, to minimize overlap between general population preferences and individual target preferences. Their findings indicated that participants could reliably estimate preferences for movies and foods, while accuracy was lower for other categories such as books and bags. This suggests that, in certain domains, participants were able to accurately estimate others’ preferences, even with very brief exposure to limited information, such as facial appearance. Given these considerations, we adopted food and movie posters as stimuli in our study, aligning with prior research demonstrating that these categories are suitable for assessing accuracy in estimating others’ preferences.

To enhance participants’ engagement in estimating the targets’ preferences, they were informed that monetary incentives would be given based on their relative performance. Notably, no participant had prior familiarity with any of the photo targets. Before the MRI scanning, participants also provided facial photographs for later use in their own personal preference estimation condition.

**Figure 1.**
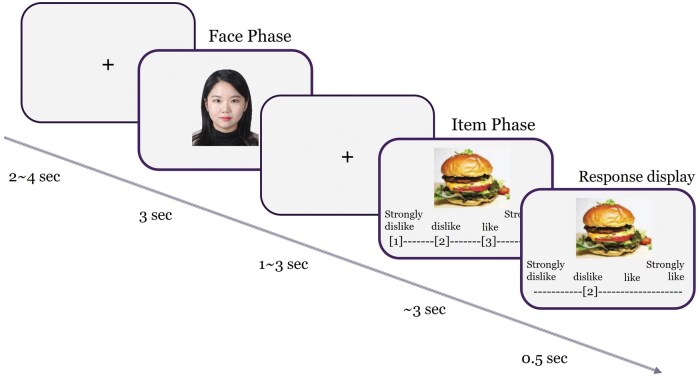
Task design. In the preference estimation task, the participants were asked to infer the target’s preference for a given item. A target was presented during the face phase for 3 s in the target-trials, and then an item was displayed. During the item phase, the participants had to perceive an item and rate how much the target would favour this specific item on a 4-point scale. When the participants answered, their response was shown on the screen for 0.5 s.

During the single MRI session, which lasted approximately 18min, participants performed the preference estimation task (Fig. 1), with trials presented in event-related paradigms. Each trial began with a 3-second display of a facial photo (either of the participant self or a target), followed by a jittered fixation interval lasting 2–4seconds. Subsequently, an item was displayed, and participants provided their own preference (self-trial) or guessed the targets’ preference (target-trial) for the depicted item using a 4-point Likert scale (from ‘strongly dislike’ to ‘strongly like’) within a 3-second response window. Immediately after the response, participant’s choice was displayed on the screen for 0.5seconds. It is important to note that the ‘participant’s choice’ here refers to the predictions and preference inferences made solely by the participants, rather than feedback on the actual preferences of the targets. No information about the actual preferences of the 8 targets was provided to the participants at any point in the study. Therefore, there was no chance for participants to learn or adjust their predictions based on feedback. Each prediction was made independently on each trial, based solely on the participants’ judgement without any external information influencing subsequent estimations. The MRI session comprised 10 self-trials and 80 target-trials (i.e., facial photo (9) × item (10)). Trials were presented in a pseudo-random order. In the post-scan session, participants were debriefed about the study’s objectives and compensated with 40,000 KRW (approximately $38).

### Behavioural data analysis

**Figure 2.**
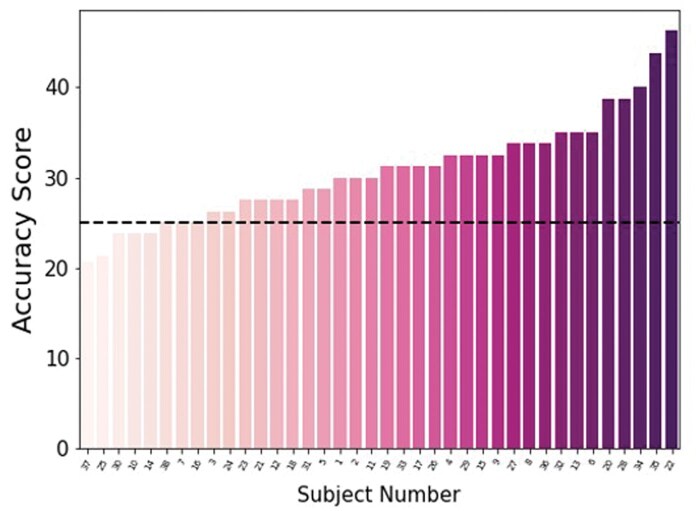
Accuracy scores (percentage) of each participant. Participants scored above the chance level (dot line) on average (*M* = 0.31, $t_{\left(37\right)}$ = 5.91, *P* < .001).

We measured preference estimation accuracy by assessing the proportion of trials where participants’ estimations precisely matched the targets’ stated preferences. Only exact matches were considered correct when calculating the accuracy score. For instance, if a target rated an item as 3 (like) and a participant estimated it as 2 (dislike), it was marked incorrect. Similarly, if a target rated an item as 3 (like) and the estimation was 4 (highly like), despite both being positive, the trial was still deemed incorrect. Overall, the accuracy of participants’ estimation was significantly above the chance level (0.25) on average (Fig. 2), meaning that participants can infer others’ preference from their faces better than random guessing (*M* = 0.31, $t_{\left(37\right)}$ = 5.91, *P* < .001). Furthermore, we conducted supplementary analyses covering various aspects, including baseline preferences for movie and food stimuli (Supplementary Fig. S1), preferences by target gender (Supplementary Fig. S2), participants’ accuracy rates for each target (Supplementary Fig. S3), accuracy differences between movie and food stimuli (Supplementary Fig. S4), and performance comparisons between high and low accuracy groups (Supplementary Fig. S5).

### fMRI data acquisition

All the neuroimaging data were collected from a 3T Siemens Trio MRI scanner (MAGNETOM Trio, A Tim System; Siemens AG, Erlangen, Germany) with a 12-channel head coil located at the Korea University Brain Imaging Center. We acquired functional images using gradient echo-planar images (EPI) with Blood Oxygenation Level-Dependent contrast (TR = 2000ms; TE = 30ms; flip angle = 90°; FOV = 240mm; 3 × 3 × 3mm in-plane resolution; 80 × 80 matrix size; and 36 slices with no gap with interleaved sequence), and high-resolution structural images (TR = 1900ms; TE = 2.52ms; flip angle = 9°; 1 × 1 × 1mm in-plane resolution; and 256 × 256 matrix size). The experiment task was presented through an MR-compatible LCD monitor mounted on a head coil (refresh rate: 60Hz; display resolution: 640 × 480 pixels; viewing angle: 30°) operating on MATLAB 2009b with Cogent 2000 stimulus presentation software.

### Image preprocessing

The functional data were preprocessed by using the default preprocessing pipelines of the CONN toolbox 2018b (www.nitrc.org/projects/conn, RRID: SCR_009550; Whitfield-Gabrieli and Nieto-Castanon 2012). The images were first realigned and unwarped, centred to (0, 0, 0) coordinates, and slice-time corrected in sequential order. The resulting images were then spatially normalized to the standard Montreal Neurological Institute 152 reference template resampled to 2mm isotropic voxels. Finally, the normalized images were then smoothed with an 8-mm full-width at half-maximum Gaussian kernel.

### Neuroimaging data analysis: Univariate approach

In the first-level analyses, we processed the neuroimaging data utilizing SPM12 software (Wellcome Department of Imaging Neuroscience, London, United Kingdom). Individual participant data were modelled using a general linear model (GLM), which incorporated five regressors: (i) the self-trial during the face phase, (ii) the target-trial during the face phase, (iii) the self-trial during the item phase, (iv) the target-trial during the item phase, and (v) button response onset. Additionally, we accounted for head-motion artefacts by including six head-motion regressors as covariates of no interest.

To identify brain regions associated with individual preference estimation performance, we performed a linear regression analysis, incorporating the contrast images derived from target-trials versus self-trials during the item phase and individual accuracy scores as covariates. A parallel analysis was applied to the data obtained during the face phase.

To precisely examine our primary hypothesis—that there exists a correlation between the activation of the dmPFC and the participants’ accuracy scores, as suggested by previous research utilizing the same paradigm (Kang et al. 2013)—we utilized a dmPFC binary mask. This mask was derived from a meta-analysis by de la Vega et al. (2016), which segmented the medial prefrontal cortex (mPFC) into nine distinct subregions based on each region’s functional coactivation maps. Our methodological choice was guided by our focused research question, leading us to opt for a univariate approach exclusively targeting the dmPFC subregion [A1], excluding other mPFC subregions from our analysis. This approach was not only aimed at confirming previous findings but also at providing a rigorous test of the role this particular subregion plays in the task at hand, thereby advancing our understanding of the functional specialization within the mPFC in the context of preference estimation. In addition to our primary objective, we sought to determine if other subregions of the mPFC played a role in the preference inference process. To this end, we conducted further analysis using an mPFC binary mask that encompasses dmPFC [A1], pgACC [A2], and vmPFC [A3].

The motivation to accurately infer others’ preferences can conflict with the motivation to express one’s own preferences. According to our model, such conflicts between motivation embedded in the vmPFC appear to be resolved through close communication with the dmPFC, leading to value adjustment (Kim 2020). Based on this hypothesis, in addition to the GLM-based fMRI data analysis, we ran a generalized psychophysiological interaction (gPPI) analysis (McLaren et al. 2012), which is particularly useful in examining task-dependent functional connectivity, to test if individuals with high accuracy in predicting others’ preferences would exhibit stronger functional connectivity between the vmPFC and dmPFC in the ‘other’ condition compared to the ‘self’ conditions. At an individual level, a voxel of interest (VOI) was extracted with the 5mm sphere around the peak (coordinates: *x* = 0, *y* = 38, *z* = *−*10) of the vmPFC cluster found in the multiple regression analysis. We utilized this VOI as a seed region. Time series were extracted from the vmPFC and served as the physiological variable, reflecting the neural activity in that region during the task. The contrasts for target- versus self-trials during the item phase served as the psychological variable. Then, the physiological and psychological variables were multiplied to create the PPI term. Finally, the individual accuracy scores were regressed with the contrast images of PPI term.

Our particular interest was the functional coupling between the mPFC subregions, with a specific focus on the vmPFC and dmPFC. To implement this analysis, we used an dmPFC binary mask [A1], consistent with the GLM-based approach above. This allowed us to explore whether the contextual connectivity strength within the mPFC increased during target-trials compared to the self-trials among the participants with higher accuracy scores.

### Neuroimaging data analysis: Multivariate approach

We utilized MVPA to identify brain regions that encapsulate inter-subject variability during the estimation of preferences based on facial feature processing. In our study, we employed inter-­subject RSA (IS-RSA; van Baar et al. 2019) to identify regions associated with variability in preference estimation accuracy. The main question was which brain regions exhibited similar activation patterns among participants with comparable accuracy scores in estimating others’ preferences.

First, we segmented the entire brain into functionally relevant regions using a predefined 200-parcel map from the Neurosynth database (https://neurosynth.org/). This map organizes the global brain atlas based on meta-analytic functional coactivation patterns. In our analysis, 183 parcels were included, omitting seventeen parcels due to their absence in our scan scope. Given prior research indicating distinct statistical properties between univariate and multivariate approaches (Jimura and Poldrack 2012, Coutanche 2013, Davis et al. 2014), we extended the ROI map initially used for univariate analysis. Importantly, MVPA captures heightened sensitivity to within-subject voxel variability but displays diminished sensitivity to inter-subject mean activation variability compared to univariate analyses (Davis et al. 2014). To understand individual differences in preference accuracy, we computed a behavioural dissimilarity matrix by calculating the absolute difference in accuracy scores among all participant dyads.

Subsequently, we constructed an inter-subject dissimilarity matrix for each of the 183 neural activation map parcels to assess inter-subject dissimilarity in mean activity patterns during the inference of the target’s preference. We used the identical contrast map as in the univariate analyses for inter-subject representational similarity analysis (IS-RSA). Of particular interest were the target-­trials compared to self-trials during the item and face phases. To identify brain regions where neural representations corresponded with behavioural inclination, we computed a nonparametric Kendall’s tau-a correlation between the parcel dissimilarity matrices and the behavioural ones (Nili et al. 2014). The significance of tau-a was validated through permutation testing. Specifically, while retaining the neural Representational Dissimilarity Matrix (RDM), we randomized the behavioural RDM 10,000 times to create a null distribution. To account for multiple comparisons, we adjusted the *P*-values using an FDR correction, and *P*-values below .01 post-adjustment indicated a significant correlation between inter-subject behavioural patterns of preference estimation accuracy and neural representation patterns of the parcel.

## Results

### Neuroimaging results: Multiple regression analysis with estimation accuracy as covariate

**Figure 3.**
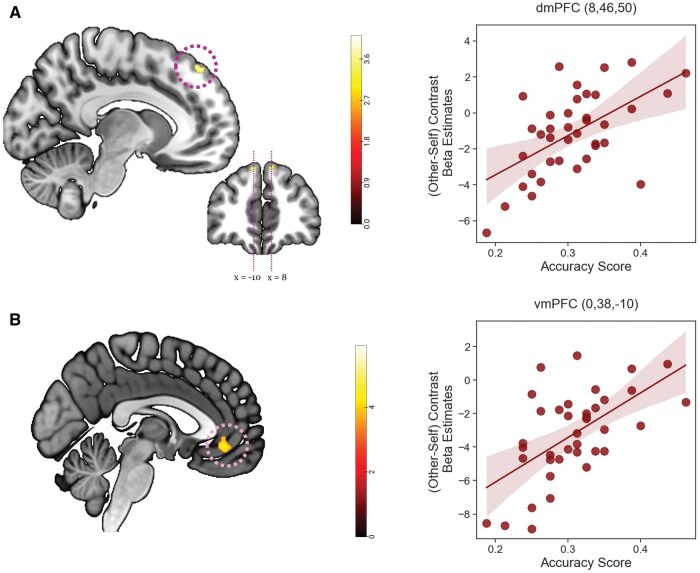
Univariate results. The mPFC activity predicts the estimation accuracy score. (A) The neural activations of the dmPFC (peak coordinates: *x* = 8, *y* = 46, *z* = 50) were positively correlated with how accurately participants guessed the targets’ actual preferences (SVC corrected, $P_{\mathrm{SVC}-\mathrm{peakFWE}}$ = 0.028) during the item phase. (B) The vmPFC (peak coordinates: *x* = 0, *y* = 38, *z* = -10) activity also positively correlated with the accuracy score (SVC corrected, $P_{\mathrm{SVC}-\mathrm{clusterFWE}}$ = 0.028) during the item phase. The scatterplot of the mPFC activations and the accuracy score is presented on the right side. The shaded area indicates the 95% confidence interval.

Our primary objective in the univariate analysis was to replicate the findings of the previous study (Kang et al. 2013), regarding the involvement of the dmPFC in accurately estimating another person’s preferences with limited information. From the mPFC subregions (de la Vega et al. 2016), we included dmPFC area [A1] as binary mask for a regression analysis. Individual contrast maps of target- vs. self- trials were regressed against individual accuracy scores as a covariate. As predicted, during the item phase, we found a significant correlation in the dmPFC: a right cluster (peak coordinates: *x* = 8, *y* = 46, *z* = 50, small volume correction (SVC) corrected, $P_{\mathrm{SVC}-\mathrm{peakFWE}}$ = 0.028) and a left cluster (peak coordinates: *x* = -10, *y* = 44, *z* = 50, small volume correction (SVC) corrected, $P_{\mathrm{SVC}-\mathrm{peakFWE}}$ = 0.038) (Fig. 3). This suggests that individuals with higher accuracy scores exhibited greater activity within these clusters during the evaluation of items for the targets compared to themselves. No significant correlation was found during the face phase.

Although the dmPFC was the primary ROI of the regression analysis, we performed additional analysis to explore whether other regions of the mPFC was engaged in accurately inferring other’s preferences. Therefore, we included other mPFC subregions, including dmPFC [A1], pgACC [A2], and vmPFC [A3], as a mask. The result revealed a significant correlation in the vmPFC (peak coordinates: *x* = 0, *y* = 38, *z* = -10, small volume correction (SVC) corrected, $P_{\mathrm{SVC}-\mathrm{clusterFWE}}$ = 0.028) during the item phase. Conversely, no significant correlation was found during the face phase. In addition, no brain regions showed significant results after correction for multiple comparisons at the whole-brain level.

### Neuroimaging results: Functional connectivity between vmPFC and dmPFC in relation to individual estimation accuracy score

**Figure 4.**
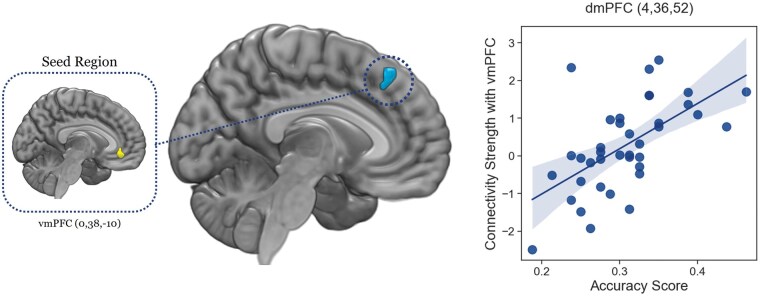
gPPI result. The functional connectivity of the vmPFC and dmPFC is linked to higher accuracy scores in estimating targets’ preferences. A heightened psychophysiological interaction with vmPFC activity was observed in the dmPFC (peak coordinates: *x* = 4, *y* = 36, *z* = 52, small volume correction (SVC) corrected, $P_{\mathrm{SVC}-\mathrm{peakFWE}}$ = 0.034) during target-trials compared to self-trials. The scatterplot on the right side illustrates the relationship between the strength of connectivity between the vmPFC and dmPFC and individual accuracy scores in estimating preferences, with the shaded area indicating the 95% confidence interval.

Multiple regression analysis revealed that not only did dmPFC but also vmPFC regions activated more strongly as the participants’ accuracy score increased. To examine the underlying rationale, we delved into the neural connectivity within mPFC subregions in relation to individual variations in preference estimation accuracy. This scrutiny pinpointed a significant cluster in the dmPFC (peak coordinates: *x* = 4, *y* = 36, *z* = 52, small volume correction (SVC) corrected, $P_{\mathrm{SVC}-\mathrm{peakFWE}}$ = 0.034, Fig. 4). Specifically, functional connectivity between the vmPFC and dmPFC intensified among the participants with higher accuracy when discerning targets’ preferences compared to when expressing their own preferences.

### Neuroimaging results: Inter-subject representational similarity analysis (is-RSA)

**Figure 5.**
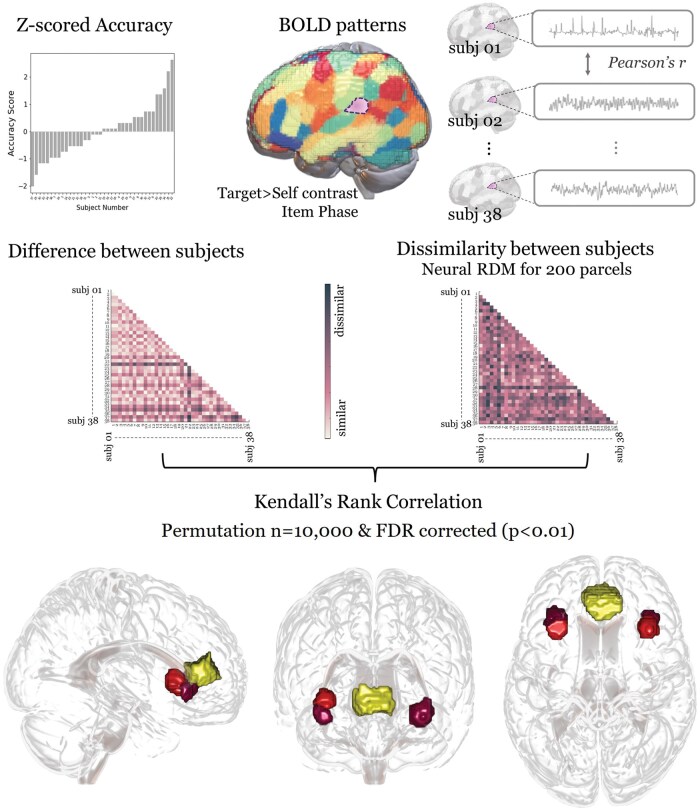
Multivariate results. Inter-subject representational similarity analysis indicates that participants with similar accuracy score exhibited similar neural patterns in brain regions including the pgACC and insula.

Significant inter-subject representational similarity effects were found in four brain parcels, during the item phase, including the ventral anterior insula (vAI), the dorsal anterior insula (dAI), and the pregenual anterior cingulate cortex (pgACC) (Fig. 5). This implies that the inter-subject dissimilarity of neural activity patterns during the estimation of others’ preferences corresponded to the inter-subject distance pattern of accuracy score. Conversely, no significant is-RSA effect was yielded during the face phase. These results indicate that individuals with similar accuracy scores shared similar multi-voxel patterns only during the item phase, not when processing the facial features of the target.

**Figure 6.**
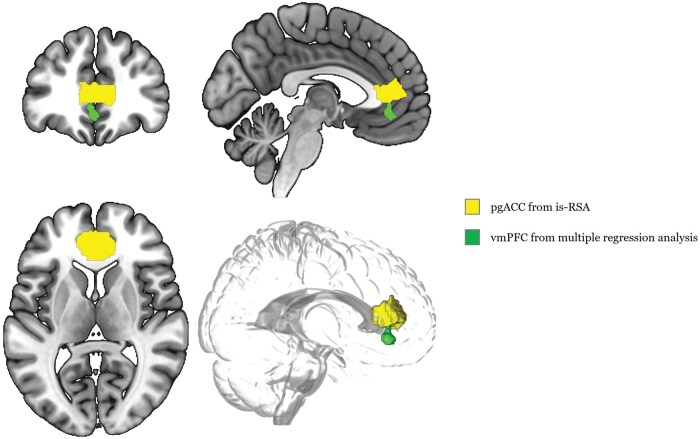
Overlay of results. The vmPFC cluster (green) identified through the univariate approach is combined with the pgACC parcel (yellow) from the multivariate approach.

Notably, the pgACC parcel identified through the is-RSA did not overlap with the vmPFC cluster obtained through the univariate approach (Fig. 6). Despite their close proximity, the absence of overlap suggests that both analytical methods can yield analogous yet distinctly separate outcomes. When using continuous measures, such as distance from the correct rating and its inverse, only the insula showed significant association with preference estimation in multivariate analysis, with no significant regions in univariate analysis. This suggests that exact accuracy may be closely linked to specific neural mechanisms, highlighting the role of precision in social inference tasks.

## Discussion

This study investigated the neural underpinnings associated with individual differences in the ability to infer others’ preferences based solely on facial cues. Consistent with the findings of Kang et al. (2013), considerable variability emerged among participants in their accuracy of estimating others’ preferences, with some individuals demonstrating adeptness while others exhibit less precision. This behavioural variation corresponded with distinct patterns observed in the neuroimaging data collected during the task. Univariate analysis reaffirmed the role of the dmPFC in preference estimation, particularly highlighting its activity during the item phase as indicative of the accuracy in inferring others’ preferences. Furthermore, multivariate analysis, particularly the results from IS-RSA, showed correlations between participants’ estimation accuracy patterns and neural activity patterns in the pgACC, ventral AI, and dorsal AI during the item phase. Collectively, these findings underscore the central role of the mPFC and insula in the process of estimating others’ preferences.

Understanding others’ minds involves recognizing their thoughts and emotions, even when these mental states are not directly observable. Traditionally, two key concepts, Theory of Mind (ToM) and empathy, have framed these social cognitive capacities. However, a recent meta-analysis study suggests that these terms have functioned as ‘umbrella terms’ encompassing various processes (Schaafsma et al. 2015, Zaki 2017). This has led to inconsistent use of terminology, with different terms being used to describe similar process and vice versa (Schurz et al. 2021). Proposals have been made for a coherent hierarchical model of social cognitive processes, positioning empathy and ToM as higher-order processes. The discussion also advocates for breaking down these processes into smaller, interdependent components through ‘deconstruction’ (Schaafsma et al. 2015), warranting further exploration of these building blocks. In response, our research aims to contribute as an integral component of this framework. Specifically, our study investigates the cognitive mechanisms involved when individuals infer others’ preferences through minimal facial cues combined with contextual information—item cues. Furthermore, we hypothesize that this cognitive building block is associated with neural activity in the mPFC and the insula.

### Roles of dorsal and ventral mPFC subregions in preference estimation

Various subregions of the mPFC, operating in coordination, play a pivotal role in social valuation. A recent model proposes a hierarchical organization of mPFC subregions, with the more dorsal region incorporating additional external sensory information from the environment to regulate the more ventral region, which processes intuitive social values based on internal bodily signals (Kim 2020).

Consistent with the previous study (Kang et al. 2013), our study reaffirmed the role of the dmPFC in estimating others’ preferences. The dmPFC is recognized as a key component of the broader mentalization network, contributing to a wide spectrum of social processes such as reasoning about other’s mental states (Mitchell 2008, Wagner et al. 2012, Wagner et al. 2016), forming impressions of others (Mitchell et al. 2005, Schiller et al. 2009), and predicting social interactions (Powers et al. 2016). Given its role in mentalization, it is reasonable to consider the dmPFC as pivotal in other’s preference estimation. Moreover, the dmPFC integrates information from various brain networks to guide thoughts and actions (Shackman et al. 2011), facilitating adaptive responses in a complex social environment.

Contrary to our initial expectations, the ventral region of the mPFC (vmPFC) also emerged as significant in accurately inferring others’ preferences. In addition, exploratory gPPI analysis revealed heightened functional coupling between the vmPFC and dmPFC in individuals with greater accuracy scores. This suggests that individuals with higher accuracy scores exhibit increased connectivity between these regions when assessing others’ preferences compared to when expressing their own preferences. Drawing from recent theories of morality and altruism (Haidt 2007, Kim 2020), we propose that this phenomenon may stem from an instrumental desire to accurately estimate others’ preferences, driven by a primal instinct for survival and reproduction. In other words, individuals may learn that precise predictions foster favourable impressions, leading to the internalization of this instrumental desire within the vmPFC. This facilitates an automatic and intuitive motivation to seek detailed information for preference estimations. This hypothesis aligns with evidence indicating that the vmPFC is involved in context-independent internalized prosocial valuation (Sul et al. 2015, Jung et al. 2018). Considering previous research indicating the association between the vmPFC and self-centred preferences (Kang et al. 2013), individuals with high accuracy in predicting others’ preferences may demonstrate increased vmPFC-dmPFC communication, integrating additional external information to resolve conflicts between the instrumental desire for predicting others’ preferences and self-centred preferences encoded within the vmPFC (Kim 2020).

### Neural representations of pgACC and insula associated with preference estimation

In this study, our *a priori* goal was to uncover the neural patterns associated with accurately estimating others’ preferences. Our results showed that individuals with similar behavioural scores exhibited comparable activity patterns in the pgACC and insula, both crucial for interoception—the perception of internal bodily sensations (Critchley and Harrison 2013, Barrett and Simmons 2015). The pgACC and AI are central components of the neural circuitry linked to predicting bodily reactions or maintaining internal equilibrium, potentially through von Economo neurons (VEN), which facilitate rapid brain-body communication (Allman et al. 2010, Fischer et al. 2016). Although this connection suggests a potential link between sensing our own internal states and accurately inferring others’ preferences, it remains unclear how the interoceptive network contributes to this process of correctly inferring others’ preferences.

The pregenual anterior cingulate cortex (pgACC), in conjunction with the anterior insula (AI), also plays a key role in the processing of social information. The pgACC is actively engaged in tracking others’ motivations (Chang et al. 2013, Apps et al. 2016), differentiating between self and other-oriented information in social interactions (Lockwood and Wittmann 2018), and processing empathy (Xu et al. 2009, Wittmann et al. 2018). The activities of the pgACC were positively linked with the ability to utilize contextual information for inferring others’ emotional states (Kim et al. 2022), and damage to the pgACC can impede social awareness and empathic capabilities (Seeley 2008). The AI, another critical region, plays a significant role in social information processing and is implicated in empathy, compassion, and various interpersonal phenomena (Lamm and Singer 2010). AI integrates multiple bodily signals to simulate internal states of the targets to generate appropriate empathic behaviours (Singer et al. 2009). Difficulties in theory of mind and hypoactivity in AI during face processing have been noted in autism spectrum disorder (Uddin and Menon 2009). In the context of social inference concerning the learning of others’ preferences, both the pgACC and AI contribute to these complex processes (Lau et al. 2020). Furthermore, individual differences in the activities of the pgACC and AI signify the degree of context dependency in estimating other’s affective states (Kim et al. 2022). The extensive involvement of the pgACC and AI in social information processing hints that they may play a coordinating role in predicting preferences in a target individual.

From birth, individuals are deeply intertwined with their primary caregivers for survival, and as they mature, they engage in a dynamic interplay with a broader social environment, shaping and being shaped by it (Atzil et al. 2018). Consequently, individuals develop internal frameworks based on societal norms and the behaviours of those around them. These frameworks shape our ‘sense of should (Theriault et al. 2021),’ guiding us to act in accordance with cultural and social standards (Constant et al. 2019). Conforming to meet others’ expectations can foster a more stable social setting, helping us conserve energy as we navigate the intricacies of social interactions. Previous research has linked the tracking of social norms, aimed at minimizing metabolic costs, to interoceptive processing (Theriault et al. 2021, Sennesh et al. 2022).

Within the framework of interoceptive predictive coding, the brain continually forms and updates models of the external environment and internal organism states (Petzschner et al. 2021, Engelen et al. 2023). Attention plays a pivotal role in modifying these models (Feldman and Friston 2010), prompting individuals to update them in response to prediction errors. Participants who excelled in this task may habitually place significant weight on their social environment, adjusting their generative models more frequently and specifically for each individual encountered. Through consistent attention and adjustment, they likely developed more refined and detailed internal models of others, providing a reliable reference point for more accurate predictions despite task’s requirement to predict the preferences of unfamiliar individuals.

This study suggests that that activation patterns in the mPFC and AI are crucial in accounting for individual differences in thin-slicing ability—the capacity to accurately predict others’ preferences based on briefly presented facial cues. Given that these regions are key components of the interoceptive neural circuitry, it is plausible that constructing an accurate predictive model of others’ preferences—by minimizing the gap between expectations and observed behaviours—requires close communication between the brain and body. Such a refined model could facilitate social interactions, reduce conflicts, and ultimately enhance social adaptability by conserving bodily resources.

### Interoceptive network in cue integration

Another noteworthy discovery in this study pertains to the neural patterns in the pgACC and insula, which exhibited variations among participants only during the item phase, not the face phase. This suggests that when facial information was presented, no regions showed neural patterns similar to those of the behavioural accuracy score, implying that estimation accuracy was not contingent upon distinct facial information processing alone. Instead, the critical determinant of preference accuracy appeared to be the integration of both facial and item information, with the insula and pgACC emerging as a pivotal region in this integration process. This finding resonates with previous study identifying the AI’s crucial role in cue integration for empathic responses and contextual dependency in ambiguous facial emotion processing (Kim et al. 2022). Conjunction analyses focusing on empathy, interoception, and social cognition have highlighted the AI as a central hub for integrating interoceptive and social information (Adolfi et al. 2017). Moreover, the communication between the pgACC and AI has been implicated in interoceptive prediction (Barrett and Simmons 2015). Consequently, we hypothesize that both the pgACC and insula play pivotal roles in interoception and social cognition, seamlessly integrating internal and external information to construct sophisticated models of the social environment relevant to an individual’s survival goals.

### Methodological considerations

This research particularly focuses on the discrepancy between univariate and multivariate analyses. While the pgACC and insula were identified as significant regions in RSA analyses, they were not found in univariate approaches. MVPA is known for its superior sensitivity in detecting subtle differences between psychological states, whereas univariate analysis is more sensitive to inter-subject variability in mean activation across voxels within a specific ROI (Davis et al. 2014, Kohoutová et al. 2020). In support of this, it has been observed that even within the same brain regions, univariate and multivariate analyses can yield opposing results (Woo et al. 2014, Kim and Kim 2021). It has been established that the magnitude of the BOLD response is sensitive to changes in the excitation-inhibition balance within cortical microcircuits, which involve pyramidal projection neurons interacting with local GABAergic interneurons, possibly reflecting mismatch or prediction error-related feedback signals (Logothetis 2008). From this perspective, the dmPFC activity observed in univariate analysis could be interpreted as reflecting the activity of local GABAergic interneurons responding to mismatches or prediction errors arising from the comparison between internal models and external information in the process of inferring others’ preferences. In contrast, the pgACC and insula activity observed in multivariate analysis may not be directly related to this preference inference process. While multivariate analysis provides valuable insights into the neural representations associated with individual differences, the lack of convergence across different analytical methods highlights the need for further investigation. Moreover, MVPA, originally developed as a predictive tool, may not always be suitable for interpreting brain function and is more complex than univariate analysis, requiring caution (Hebart and Baker 2018). Future research should employ complementary analytical approaches to better understand the neural mechanisms underlying preference estimation.

The activation patterns of the mPFC and AI were correlated with fine-grained accuracy scores, but not with categorical accuracy. Here, categorical accuracy refers to a coarser measure in which ‘like’ and ‘strongly like’ responses are collapsed into a single category, as are ‘dislike’ and ‘strongly dislike.’ This distinction emphasizes that the observed neural correlates may be more strongly linked to precise, fine-grained estimations than to broad categorical classifications.

This discrepancy between fine-grained accuracy scores and categorical accuracy scores may stem from the fact that our behavioural task required participants to choose one out of four options rather than simply indicating like versus dislike. A four-choice task likely demands more extensive information processing and mentalization than a two-choice task, potentially engaging different neural circuits. Even if the same neural circuits are involved, the level and pattern of activation might vary. Future research should investigate whether a like versus dislike task would reveal correlations between the activation patterns of the mPFC and AI would and categorical accuracy. Furthermore, this distinction aligns with hierarchical models of social cognition, which propose that higher-order cognitive processes involve complex and abstract reasoning, whereas lower-order processes rely on faster, heuristic-based decision-making (Schurz et al. 2021). Given that fine-grained judgements require detailed consideration of individuating information rather than broad categorical assumptions, they are more likely to engage neural regions associated with higher-order social inference. In contrast, categorical classification often relies on heuristic processing, allowing for rapid but less nuanced judgments. Our findings, therefore, suggest that the involvement of the mPFC and AI in fine-grained accuracy may reflect the increased cognitive demands of more effortful social inference.

### Limitations

This study has a limitation that the participant pool consisted exclusively of female participants. This choice was made to control for potential gender differences in social cognition and preference estimation, as prior studies have indicated such disparities (Carney et al. 2007). However, this limits the generalizability of our findings to a broader population, including males or mixed-gender groups. Additionally, participants may have inferred the targets’ preferences based on various pieces of information that can be extracted from their faces (e.g., BMI, facial expression). Future research should aim to include a more diverse sample to improve generalizability and examine which specific facial cues contribute to preference estimation and to what extent.

Furthermore, while our Bayesian post hoc power analysis suggests that the study was likely well-powered to detect the observed effects, we acknowledge that post hoc power calculations based on observed data may provide biased estimates of actual power (Heinsberg and Weeks 2022). These results should therefore be interpreted with caution. Nonetheless, we believe that our significant findings provide credible initial evidence for reported effects. In line with recent study (Lengersdorff and Lamm 2025), we suggest that statistically significant results can retain evidential value even when derived from studies that may not meet traditional power thresholds. Future research should conduct a priori power analyses based on the effect sizes observed in the present study to firmly establish the robustness and replicability of these findings.

## Conclusion

In conclusion, this study delved into individual variability in accurately estimating the preferences of strangers based on minimal information. Consistent with prior research, participants overall surpassed chance level estimations, demonstrating a wide range of accuracy scores, with the dmPFC playing a significant role. Notable, activity patterns within the interoceptive network, particularly the pgACC and insula, correlated with similar accuracy scores during the item phase. These findings underscore the neural signatures of individual differences in accurately assessing others’ preferences and tailoring estimations to specific targets rather than relying on broad generalizations. We propose that the ability to rapidly infer others’ preferences from sparse information serves as a foundational, lower-order process contributing to the more complex, higher-order processes facilitating social cognition. However, further research is needed to elucidate the precise mechanisms of this process in more detail.

## Supplementary Material

nsaf051_Supplementary_Data

## Data Availability

To promote transparency and reproducibility, we are committed to sharing our data and analysis scripts upon request. Researchers interested in accessing our data and scripts may contact the corresponding author. We will ensure that all shared materials adhere to ethical guidelines and institutional policies to protect participant confidentiality.

## References

[nsaf051-B1] Adolfi F , Couto B, Richter F et al Convergence of interoception, emotion, and social cognition: a twofold fMRI meta-analysis and lesion approach. Cortex 2017;88:124–42. 28088652 10.1016/j.cortex.2016.12.019

[nsaf051-B2] Albrechtsen JS , Meissner CA, Susa KJ. Can intuition improve deception detection performance? J Exp Soc Psychol 2009;45:1052–5.

[nsaf051-B3] Allman JM , Tetreault NA, Hakeem AY et al The von economo neurons in frontoinsular and anterior cingulate cortex in great apes and humans. Brain Struct Funct 2010;214:495–517. 20512377 10.1007/s00429-010-0254-0

[nsaf051-B4] Ambady N , Rosenthal R. Thin slices of expressive behavior as predictors of interpersonal consequences: a meta-analysis. Psychol Bull 1992;111:256–74. 10.1037/0033-2909.111.2.256

[nsaf051-B5] Amodio DM , Frith CD. Meeting of minds: the medial frontal cortex and social cognition. Nat Rev Neurosci 2006;7:268–77. 16552413 10.1038/nrn1884

[nsaf051-B6] Apps MA , Rushworth MF, Chang SW. The anterior cingulate gyrus and social cognition: tracking the motivation of others. Neuron 2016;90:692–707. 27196973 10.1016/j.neuron.2016.04.018 PMC4885021

[nsaf051-B7] Atzil S , Gao W, Fradkin I et al Growing a social brain. Nature Human Behaviour 2018;2:624–36. 10.1038/s41562-018-0384-6 31346259

[nsaf051-B8] Barrett LF , Simmons WK. Interoceptive predictions in the brain. Nat Rev Neurosci 2015;16:419–29. 26016744 10.1038/nrn3950 PMC4731102

[nsaf051-B9] Carney DR , Colvin CR, Hall JA. A thin slice perspective on the accuracy of first impressions. J Res Pers 2007;41:1054–72.

[nsaf051-B10] Chang SW , Gariépy J-F, Platt ML. Neuronal reference frames for social decisions in primate frontal cortex. Nat Neurosci 2013;16:243–50. 23263442 10.1038/nn.3287 PMC3557617

[nsaf051-B11] Chwe AH , Freeman JB. Trustworthiness of crowds is gleaned in half a second. Soc Psychol Personal Sci 2023;15:351–59.

[nsaf051-B12] Constant A , Ramstead MJ, Veissière SP et al Regimes of expectations: an active inference model of social conformity and human decision making. Front Psychol 2019;10:679. 30988668 10.3389/fpsyg.2019.00679 PMC6452780

[nsaf051-B13] Contreras-Huerta LS , Coll M-P, Bird G et al Neural representations of vicarious rewards are linked to interoception and prosocial behaviour. Neuroimage 2023;269:119881. 36702212 10.1016/j.neuroimage.2023.119881

[nsaf051-B14] Coutanche MN. Distinguishing multi-voxel patterns and mean activation: why, how, and what does it tell us? Cogn Affect Behav Neurosci 2013;13:667–73. 23857415 10.3758/s13415-013-0186-2

[nsaf051-B15] Critchley HD , Harrison NA. Visceral influences on brain and behavior. Neuron 2013;77:624–38. 23439117 10.1016/j.neuron.2013.02.008

[nsaf051-B16] Davis T , LaRocque KF, Mumford JA et al What do differences between multi-voxel and univariate analysis mean? How subject-, voxel-, and trial-level variance impact fMRI analysis. Neuroimage 2014;97:271–83. 24768930 10.1016/j.neuroimage.2014.04.037 PMC4115449

[nsaf051-B17] De Freitas J , Hafri A. Moral thin-slicing: forming moral impressions from a brief glance. J Exp Soc Psychol 2024;112:104588.

[nsaf051-B18] de la Vega A , Chang LJ, Banich MT et al Large-scale meta-analysis of human medial frontal cortex reveals tripartite functional organization. J Neurosci 2016;36:6553–62. 27307242 10.1523/JNEUROSCI.4402-15.2016 PMC5015787

[nsaf051-B19] Du H , Wang L. A bayesian power analysis procedure considering uncertainty in effect size estimates from a meta-analysis. Multivariate Behav Res 2016;51:589–605. 27485763 10.1080/00273171.2016.1191324

[nsaf051-B20] Dvash J , Shamay-Tsoory SG. Theory of mind and empathy as multidimensional constructs: Neurological foundations. Top Lang Disord 2014;34:282–95.

[nsaf051-B21] Eggleston CM , Wilson TD, Lee M et al Predicting what we will like: Asking a stranger can be as good as asking a friend. Organ Behav Hum Decis Processes 2015;128:1–10.

[nsaf051-B22] Engelen T , Solcà M, Tallon-Baudry C. Interoceptive rhythms in the brain. Nat Neurosci 2023;26:1670–84. 37697110 10.1038/s41593-023-01425-1

[nsaf051-B23] Feldman H , Friston KJ. Attention, uncertainty, and free-energy. Front Hum Neurosci 2010;4:215. 21160551 10.3389/fnhum.2010.00215 PMC3001758

[nsaf051-B24] Fischer DB , Boes AD, Demertzi A et al A human brain network derived from coma-causing brainstem lesions. Neurology 2016;87:2427–34. 27815400 10.1212/WNL.0000000000003404 PMC5177681

[nsaf051-B25] Frith CD , Frith U. Mechanisms of social cognition. Annu Rev Psychol 2012;63:287–313. 21838544 10.1146/annurev-psych-120710-100449

[nsaf051-B26] Gosling SD , Ko SJ, Mannarelli T et al A room with a cue: personality judgments based on offices and bedrooms. J Pers Soc Psychol 2002;82:379–98. 11902623 10.1037//0022-3514.82.3.379

[nsaf051-B27] Haidt J. The new synthesis in moral psychology. Science 2007;316:998–1002. 17510357 10.1126/science.1137651

[nsaf051-B28] Hebart MN , Baker CI. Deconstructing multivariate decoding for the study of brain function. Neuroimage 2018;180:4–18. 28782682 10.1016/j.neuroimage.2017.08.005 PMC5797513

[nsaf051-B29] Heinsberg LW , Weeks DE. Post hoc power is not informative. Genet Epidemiol 2022;46:390–4. 35642557 10.1002/gepi.22464 PMC9452450

[nsaf051-B30] Jaksic C , Schlegel K. Accuracy in judging others’ personalities: the role of emotion recognition, emotion understanding, and trait emotional intelligence. J Intell 2020;8:34. 32961916 10.3390/jintelligence8030034 PMC7555973

[nsaf051-B31] Jimura K , Poldrack RA. Analyses of regional-average activation and multivoxel pattern information tell complementary stories. Neuropsychologia 2012;50:544–52. 22100534 10.1016/j.neuropsychologia.2011.11.007

[nsaf051-B32] Jones AL , Kramer RS. Facial first impressions form two clusters representing approach-avoidance. Cogn Psychol 2021;126:101387. 33964592 10.1016/j.cogpsych.2021.101387

[nsaf051-B33] Jung D , Sul S, Lee M et al Social observation increases functional segregation between MPFC subregions predicting prosocial consumer decisions. Sci Rep 2018;8:3368. 29463816 10.1038/s41598-018-21449-z PMC5820324

[nsaf051-B34] Kang P , Lee J, Sul S et al Dorsomedial prefrontal cortex activity predicts the accuracy in estimating others’ preferences. Front Hum Neurosci 2013;7:686. 24324419 10.3389/fnhum.2013.00686 PMC3840299

[nsaf051-B35] Kim H. Stability or plasticity?–a hierarchical allostatic regulation model of medial prefrontal cortex function for social valuation. Front Neurosci 2020;14:281. 32296303 10.3389/fnins.2020.00281 PMC7138052

[nsaf051-B36] Kim J , Kim H. Neural representation in MPFC reveals hidden selfish motivation in white lies. J Neurosci 2021;41:5937–46. 34059555 10.1523/JNEUROSCI.0088-21.2021 PMC8265801

[nsaf051-B37] Kim KI , Jung WH, Woo C-W et al Neural signatures of individual variability in context-dependent perception of ambiguous facial expression. Neuroimage 2022;258:119355. 35660000 10.1016/j.neuroimage.2022.119355

[nsaf051-B38] Kohoutová L , Heo J, Cha S et al Toward a unified framework for interpreting machine-learning models in neuroimaging. Nat Protoc 2020;15:1399–435. 32203486 10.1038/s41596-019-0289-5 PMC9533325

[nsaf051-B39] Kragel PA , Han X, Kraynak TE et al Functional MRI can be highly reliable, but it depends on what you measure: a commentary on Elliott et al.(2020). Psychol Sci 2021;32:622–6. 33685310 10.1177/0956797621989730 PMC8258303

[nsaf051-B40] Kraus MW , Keltner D. Signs of socioeconomic status: a thin-slicing approach. Psychological Science 2009;20:99–106. 19076316 10.1111/j.1467-9280.2008.02251.x

[nsaf051-B41] Krishnan A , Woo C-W, Chang LJ et al Somatic and vicarious pain are represented by dissociable multivariate brain patterns. Elife 2016;5:e15166. 27296895 10.7554/eLife.15166 PMC4907690

[nsaf051-B42] Kruschke JK , Liddell TM. The Bayesian new statistics: Hypothesis testing, estimation, meta-analysis, and power analysis from a Bayesian perspective. Psychon Bull Rev 2018;25:178–206. 28176294 10.3758/s13423-016-1221-4

[nsaf051-B43] Lamm C , Decety J, Singer T. Meta-analytic evidence for common and distinct neural networks associated with directly experienced pain and empathy for pain. Neuroimage 2011;54:2492–502. 20946964 10.1016/j.neuroimage.2010.10.014

[nsaf051-B44] Lamm C , Singer T. The role of anterior insular cortex in social emotions. Brain Struct Funct 2010;214:579–91. 20428887 10.1007/s00429-010-0251-3

[nsaf051-B45] Lau T , Gershman SJ, Cikara M. Social structure learning in human anterior insula. Elife 2020;9:e53162. 32067635 10.7554/eLife.53162 PMC7136019

[nsaf051-B46] Lengersdorff LL , Lamm C. With low power comes low credibility? Toward a principled critique of results from underpowered tests. Adv Methods Pract Psychol Sci 2025;8:25152459241296397.

[nsaf051-B47] Lockwood PL , Wittmann MK. Ventral anterior cingulate cortex and social decision-making. Neurosci Biobehav Rev 2018;92:187–91. 29886177 10.1016/j.neubiorev.2018.05.030 PMC7611523

[nsaf051-B48] Logothetis NK. What we can do and what we cannot do with fMRI. Nature 2008;453:869–78. 18548064 10.1038/nature06976

[nsaf051-B49] McLaren DG , Ries ML, Xu G et al A generalized form of context-dependent psychophysiological interactions (gPPI): a comparison to standard approaches. Neuroimage 2012;61:1277–86. 22484411 10.1016/j.neuroimage.2012.03.068 PMC3376181

[nsaf051-B50] Mitchell JP. Contributions of functional neuroimaging to the study of social cognition. Curr Dir Psychol Sci 2008;17:142–6.

[nsaf051-B51] Mitchell JP , Macrae CN, Banaji MR. Forming impressions of people versus inanimate objects: social-cognitive processing in the medial prefrontal cortex. Neuroimage 2005;26:251–7. 15862225 10.1016/j.neuroimage.2005.01.031

[nsaf051-B52] Nili H , Wingfield C, Walther A et al A toolbox for representational similarity analysis. PLoS Comput Biol 2014;10:e1003553. 24743308 10.1371/journal.pcbi.1003553 PMC3990488

[nsaf051-B53] Norman KA , Polyn SM, Detre GJ et al Beyond mind-reading: multi-voxel pattern analysis of fMRI data. Trends Cogn Sci 2006;10:424–30. 16899397 10.1016/j.tics.2006.07.005

[nsaf051-B54] North MS , Todorov A, Osherson DN. Inferring the preferences of others from spontaneous, low-emotional facial expressions. J Exp Soc Psychol 2010;46:1109–13.

[nsaf051-B55] North MS , Todorov A, Osherson DN. Accuracy of inferring self-and other-preferences from spontaneous facial expressions. J Nonverbal Behav 2012;36:227–33.

[nsaf051-B56] Park J , Kim H, Sohn J-W et al EEG beta oscillations in the temporoparietal area related to the accuracy in estimating others’ preference. Front Hum Neurosci 2018;12:43. 29479312 10.3389/fnhum.2018.00043 PMC5811502

[nsaf051-B57] Pennebaker JW , King LA. Linguistic styles: Language use as an individual difference. J Pers Soc Psychol 1999;77:1296–312. 10.1037/0022-3514.77.6.1296 10626371

[nsaf051-B58] Petzschner FH , Garfinkel SN, Paulus MP et al Computational models of interoception and body regulation. Trends Neurosci 2021;44:63–76. 33378658 10.1016/j.tins.2020.09.012 PMC8109616

[nsaf051-B59] Pollmann MM , Scheibehenne B. An information theory account of preference prediction accuracy. J Consum Psychol 2015;25:286–95.

[nsaf051-B60] Powers KE , Chavez RS, Heatherton TF. Individual differences in response of dorsomedial prefrontal cortex predict daily social behavior. Soc Cogn Affect Neurosci 2016;11:121–6. 26206505 10.1093/scan/nsv096 PMC4692321

[nsaf051-B61] Quesque F , Rossetti Y. What do theory-of-mind tasks actually measure? Theory and practice. Perspect Psychol Sci 2020;15:384–96. 32069168 10.1177/1745691619896607

[nsaf051-B62] Rule NO , Ambady N. Brief exposures: Male sexual orientation is accurately perceived at 50 ms. J Exp Soc Psychol 2008a;44:1100–5.

[nsaf051-B63] Rule NO , Ambady N. The face of success: Inferences from chief executive officers’ appearance predict company profits. Psychol Sci 2008b;19:109–11. 18271856 10.1111/j.1467-9280.2008.02054.x

[nsaf051-B64] Schaafsma SM , Pfaff DW, Spunt RP et al Deconstructing and reconstructing theory of mind. Trends Cogn Sci 2015;19:65–72. 25496670 10.1016/j.tics.2014.11.007 PMC4314437

[nsaf051-B65] Schiller D , Freeman JB, Mitchell JP et al A neural mechanism of first impressions. Nat Neurosci 2009;12:508–14. 19270690 10.1038/nn.2278

[nsaf051-B66] Schnell K , Bluschke S, Konradt B et al Functional relations of empathy and mentalizing: an fMRI study on the neural basis of cognitive empathy. NeuroImage 2011;54:1743–54. 20728556 10.1016/j.neuroimage.2010.08.024

[nsaf051-B67] Schurz M , Radua J, Aichhorn M et al Fractionating theory of mind: a meta-analysis of functional brain imaging studies. Neurosci Biobehav Rev 2014;42:9–34. 24486722 10.1016/j.neubiorev.2014.01.009

[nsaf051-B68] Schurz M , Radua J, Tholen MG et al Toward a hierarchical model of social cognition: a neuroimaging meta-analysis and integrative review of empathy and theory of mind. Psychol Bull 2021;147:293–327. 33151703 10.1037/bul0000303

[nsaf051-B69] Seeley WW. Selective functional, regional, and neuronal vulnerability in frontotemporal dementia. Curr Opin Neurol 2008;21:701–7. 18989116 10.1097/WCO.0b013e3283168e2d PMC2909835

[nsaf051-B70] Sennesh E , Theriault J, Brooks D et al Interoception as modeling, allostasis as control. Biol Psychol 2022;167:108242. 34942287 10.1016/j.biopsycho.2021.108242 PMC9270659

[nsaf051-B71] Seo H , Cai X, Donahue CH et al Neural correlates of strategic reasoning during competitive games. Science 2014;346:340–3. 25236468 10.1126/science.1256254 PMC4201877

[nsaf051-B72] Shackman AJ , Salomons TV, Slagter HA et al The integration of negative affect, pain and cognitive control in the cingulate cortex. Nat Rev Neurosci 2011;12:154–67. 21331082 10.1038/nrn2994 PMC3044650

[nsaf051-B73] Singer T , Critchley HD, Preuschoff K. A common role of insula in feelings, empathy and uncertainty. Trends Cogn Sci 2009;13:334–40. 19643659 10.1016/j.tics.2009.05.001

[nsaf051-B74] Sul S , Tobler PN, Hein G et al Spatial gradient in value representation along the medial prefrontal cortex reflects individual differences in prosociality. Proc Natl Acad Sci U S A 2015;112:7851–6. 26056280 10.1073/pnas.1423895112 PMC4485092

[nsaf051-B75] Theriault JE , Young L, Barrett LF. The sense of should: a biologically-based framework for modeling social pressure. Phys Life Rev 2021;36:100–36. 32008953 10.1016/j.plrev.2020.01.004 PMC8645214

[nsaf051-B76] Todorov A , Mandisodza AN, Goren A et al Inferences of competence from faces predict election outcomes. Science 2005;308:1623–6. 15947187 10.1126/science.1110589

[nsaf051-B77] Tognetti A , Berticat C, Raymond M et al Is cooperativeness readable in static facial features? An inter-cultural approach. Evol Hum Behav 2013;34:427–32.

[nsaf051-B78] Uddin LQ , Menon V. The anterior insula in autism: under-connected and under-examined. Neurosci Biobehav Rev 2009;33:1198–203. 19538989 10.1016/j.neubiorev.2009.06.002 PMC2743776

[nsaf051-B79] van Baar JM , Chang LJ, Sanfey AG. The computational and neural substrates of moral strategies in social decision-making. Nat Commun 2019;10:1483. 30940815 10.1038/s41467-019-09161-6 PMC6445121

[nsaf051-B80] Van’t Wout M , Sanfey AG. Friend or foe: the effect of implicit trustworthiness judgments in social decision-making. Cognition 2008;108:796–803. 18721917 10.1016/j.cognition.2008.07.002

[nsaf051-B81] Vijayakumar S , Hartstra E, Mars RB et al Neural mechanisms of predicting individual preferences based on group membership. Soc Cogn Affect Neurosci 2021;16:1006–17. 33025007 10.1093/scan/nsaa136 PMC8421698

[nsaf051-B82] Wagner DD , Haxby JV, Heatherton TF. The representation of self and person knowledge in the medial prefrontal cortex. Wiley Interdiscip Rev Cogn Sci 2012;3:451–70. 22712038 10.1002/wcs.1183 PMC3375705

[nsaf051-B83] Wagner DD , Kelley WM, Haxby JV et al The dorsal medial prefrontal cortex responds preferentially to social interactions during natural viewing. J Neurosci 2016;36:6917–25. 27358450 10.1523/JNEUROSCI.4220-15.2016 PMC4926239

[nsaf051-B84] Wake SJ , Izuma K. A common neural code for social and monetary rewards in the human striatum. Soc Cogn Affect Neurosci 2017;12:1558–64. 28985408 10.1093/scan/nsx092 PMC5647806

[nsaf051-B85] Whitfield-Gabrieli S , Nieto-Castanon A. Conn: a functional connectivity toolbox for correlated and anticorrelated brain networks. Brain Connect 2012;2:125–41. 22642651 10.1089/brain.2012.0073

[nsaf051-B86] Willis J , Todorov A. First impressions: Making up your mind after a 100-ms exposure to a face. Psychol Sci 2006;17:592–8. 16866745 10.1111/j.1467-9280.2006.01750.x

[nsaf051-B87] Wittmann MK , Lockwood PL, Rushworth MF. Neural mechanisms of social cognition in primates. Annu Rev Neurosci 2018;41:99–118. 29561702 10.1146/annurev-neuro-080317-061450 PMC7116801

[nsaf051-B88] Woo C-W , Koban L, Kross E et al Separate neural representations for physical pain and social rejection. Nat Commun 2014;5:5380–12. 25400102 10.1038/ncomms6380 PMC4285151

[nsaf051-B89] Xu X , Zuo X, Wang X et al Do you feel my pain? Racial group membership modulates empathic neural responses. J Neurosci 2009;29:8525–9. 19571143 10.1523/JNEUROSCI.2418-09.2009 PMC6665679

[nsaf051-B90] Zaki J. Moving beyond stereotypes of empathy. Trends Cogn Sci 2017;21:59–60. 28040334 10.1016/j.tics.2016.12.004

[nsaf051-B91] Zebrowitz LA , Hall JA, Murphy NA et al Looking smart and looking good: Facial cues to intelligence and their origins. Pers Soc Psychol Bull 2002;28:238–49.

