# Supplementary material for: Neural representations in mPFC and insula encode individual differences in estimating others’ preferences
Source: Soc Cogn Affect Neurosci. 2025 May 14;20(1):nsaf051. doi: 10.1093/scan/nsaf051 (PMC12380472; doi:10.1093/scan/nsaf051)
Supplement: nsaf051_Supplementary_Data [file nsaf051_supplementary_data.docx]

Neural representations in MPFC and insula encode individual differences in estimating others’ preferences

Hyeran Kang^†^, Kun Il Kim^†^, Jinhee Kim and Hackjin Kim*

^†: these authors contributed equally^

Laboratory of Social and Decision Neuroscience

Department of Psychology

Korea University, Seoul, Republic of Korea

***Correspondence**

Hackjin Kim, Ph.D.

School of Psychology

Korea University

145 Anam-ro, Seongbuk-gu, Seoul, 02841, Republic of Korea

hackjinkim@korea.ac.kr


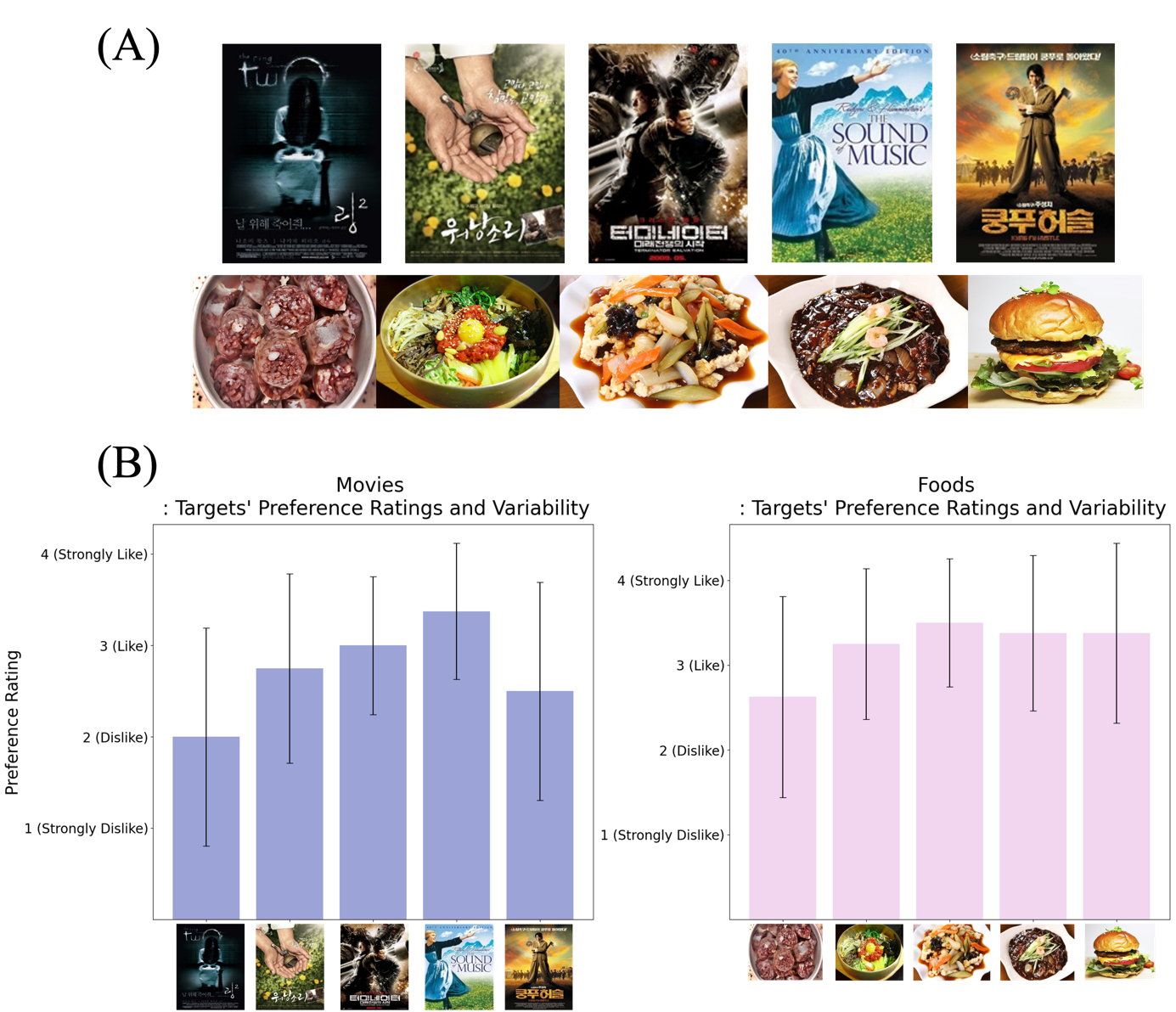


**Figure S1 | Movie and Food Stimuli with Baseline Preferences**

(A) Actual set of stimuli used in the study, including five movie posters and five food items. These items were used for participants to estimate the preferences of each target individual by observing their facial features.

(B) Bar graph representing the actual preference ratings of the targets for each item on a 4-point scale (1 = Strongly Dislike, 4 = Strongly Like), averaged across all target individuals. The left graph shows the mean preference ratings and variability (standard deviation) for the movie stimuli, while the right graph presents the same information for the food stimuli. Error bars indicate the standard deviation, illustrating the variability in how targets rated each item.


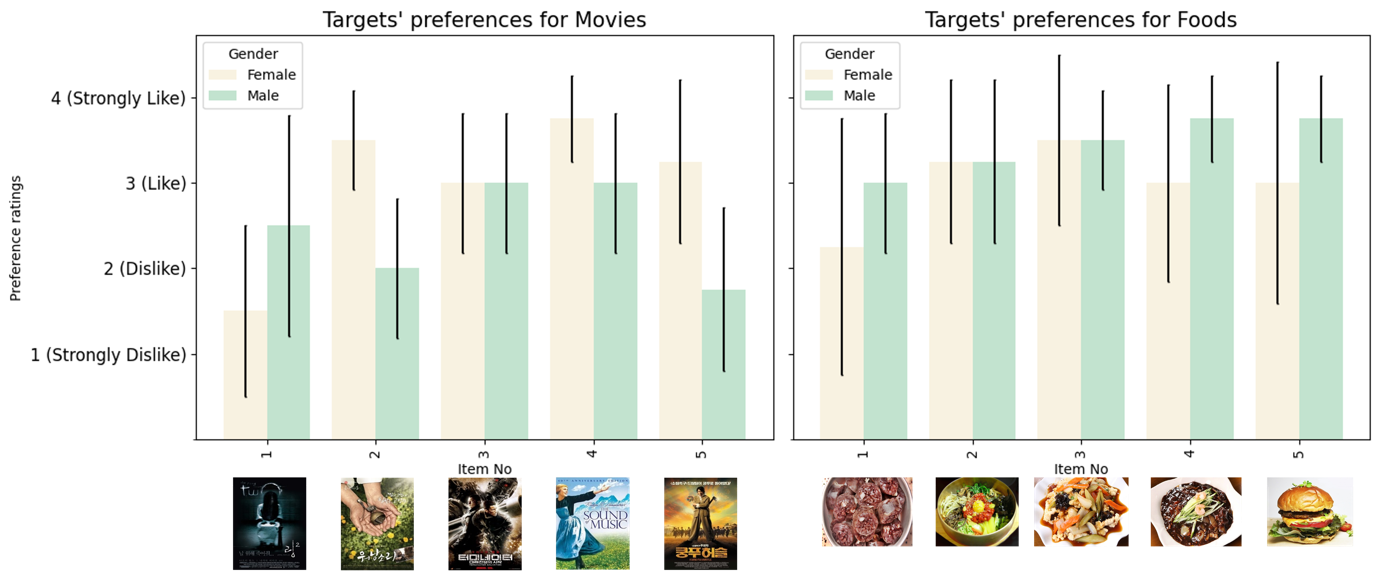


**Figure S2 | Targets’ Preferences for Movies and Foods by Gender**

This figure builds upon the baseline preference information provided in Figure S1, further breaking down the data by the gender of the targets. The left graph illustrates the mean preference ratings for each movie item, separated by male (green) and female (beige) targets, while the right graph presents the mean preference ratings for each food item. Error bars represent the standard deviation, highlighting the variability in preference ratings among the targets.

Our analysis revealed no statistically significant differences in overall preference ratings between male and female targets. Male targets' overall preferences (M = 2.95, SD = 0.99) and female targets' overall preferences (M = 3.00, SD = 1.11) were comparable (t(6) = -0.21, p > 0.05). In the movie category, male targets had a mean preference rating of 2.45 (SD = 1.00), while female targets had a mean of 3.00 (SD = 1.08; t(6) = -1.68, p > 0.05). Similarly, in the food category, male targets had a mean of 3.45 (SD = 0.69) compared to female targets' mean of 3.00 (SD = 1.17; t(6) = 1.48, p > 0.05).

In addition, we examined each item within these categories to identify any specific items that showed gender-based differences in preference. Our analysis revealed that, among the movie stimuli, only Item 2 showed a significant gender difference, with female targets (M = 3.5, SD = 0.58) showing a higher preference for this movie compared to male targets (M = 2.0, SD = 0.82; t(6) = 3.00, p = 0.024). No significant gender differences were found for any items in the food category.

These findings suggest that the stimuli used in our study were generally not subject to gender-based stereotypes. Consequently, these results imply that participants predicted others' preferences based on subtle visual cues revealed in the faces rather than relying on simple stereotypes linking specific genders to item preferences.


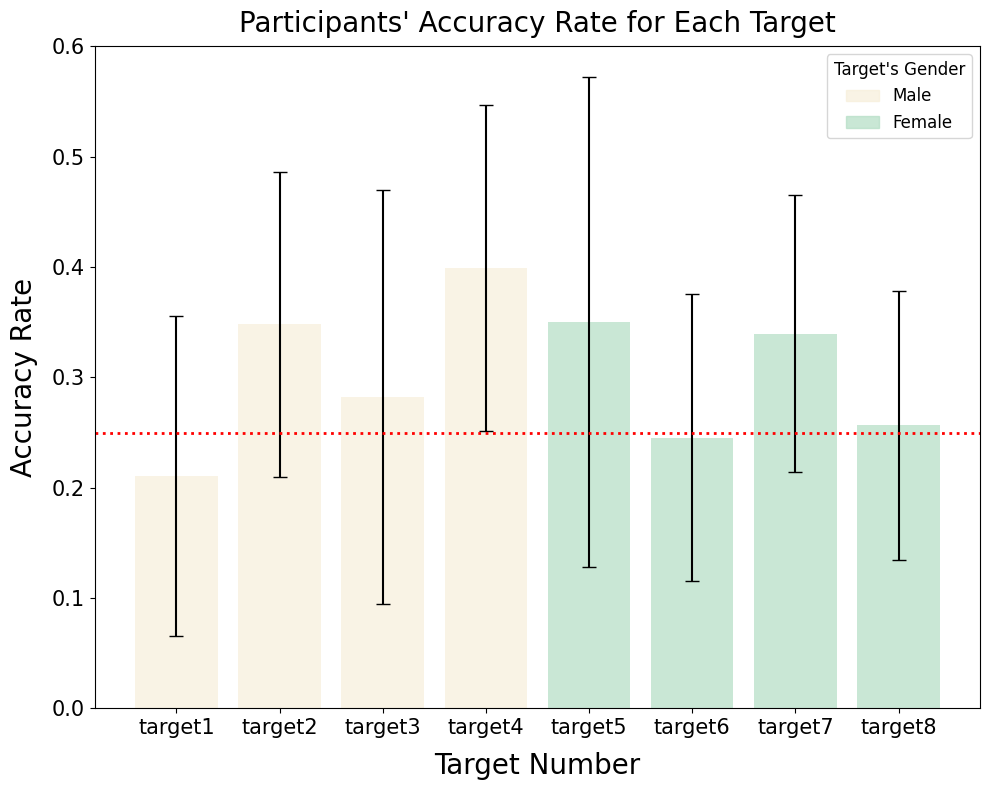


**Figure S3 | Participants’ Overall Accuracy Rate in Estimating Targets’ Preferences**

This figure shows the accuracy rates of participants when estimating the preferences of each target individual. The bars represent the average accuracy rate for each target, with error bars indicating the standard deviation, reflecting variability in participants’ accuracy. Targets are categorized by gender, with male targets shown in beige and female targets shown in green. Red dotted line indicates the chance performance level (0.25).

**Specific Details on Targets**

The targets in our study were of mixed gender (4 females and 4 males), all undergraduate students, and all were Korean. To ensure diversity and neutrality while minimizing prior familiarity, we used the paradigm developed by Kang et al. (2013), selecting four male and four female targets based on visual dissimilarity and distinct preference patterns. Our analysis of gender differences in participants’ accuracy revealed no statistically significant effect (*t*(37) = 0.85, *p* = 0.402), with accuracy rates similar for male (*M* = 0.31, *SD* = 0.07) and female targets (*M* = 0.30, *SD* = 0.08). This indicates that female participants did not perform better for female targets or worse for male targets.


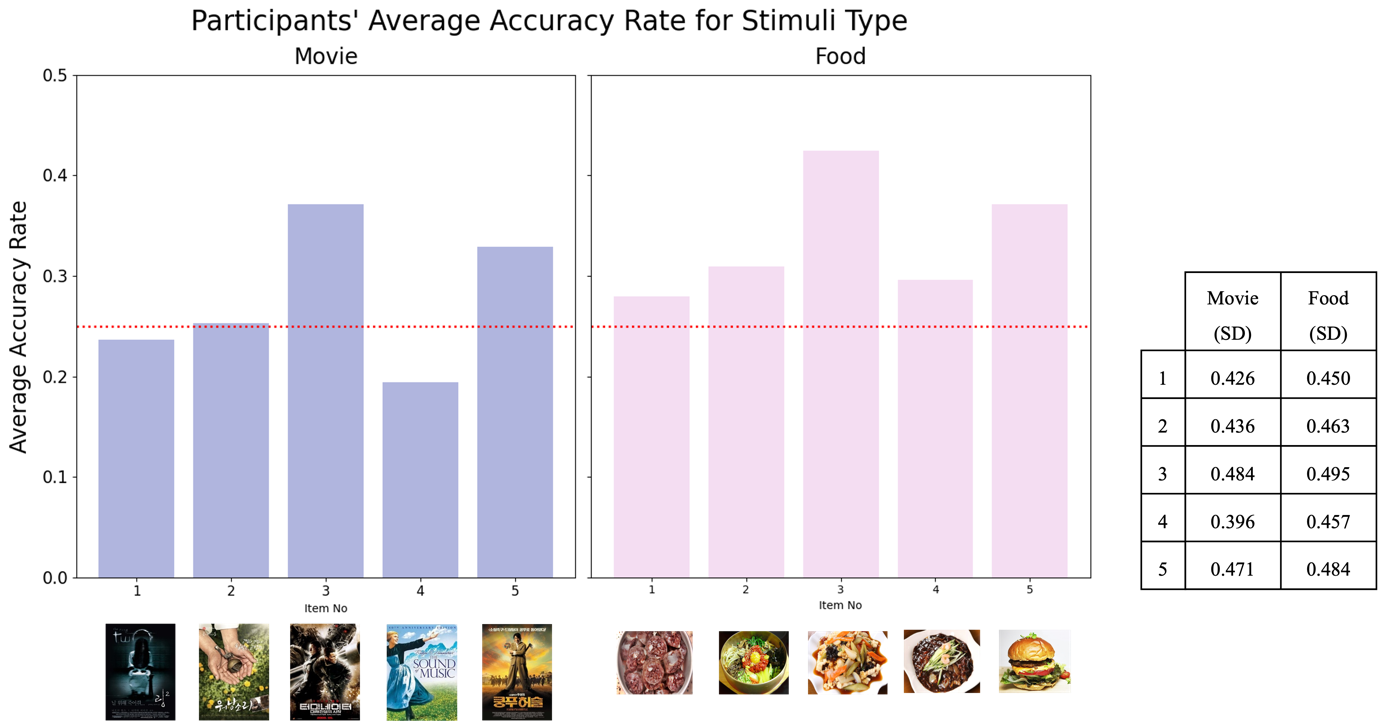


**Figure S4 | Participants’ Overall Accuracy Rate for Movie and Food Stimuli**

This figure presents the average correct rate for participants’ estimates of targets’ preferences, separately for movie and food stimuli. The left panel shows the mean correct rate for each movie item, while the right panel shows the mean correct rate for each food item. To avoid cluttering the figure, we did not include the standard deviation (SD) values directly on the bar graphs; instead, these are provided in a table on the right. The SD values were notably large, emphasizing the variability in participants’ performance across different items. Red dotted line indicates the chance performance level (0.25).

In contrast to S1, which displayed the actual preferences of each target, this figure focuses on participants’ accuracy rates for each specific stimulus, highlighting the items that were easier or harder to predict.

**Specific Details on Item Type**

Statistical analysis revealed a significant difference in accuracy between the two types of stimuli (*t*(37) = -3.096, *p* < 0.05). Participants were more accurate when estimating preferences for food items (M = 0.34, SD = 0.099) compared to movie items (M = 0.28, SD = 0.065). Given these significant behavioral differences, we also explored whether distinct neural patterns emerged by analyzing food and movie items separately in the GLM and constructing corresponding contrasts. The results indicated that the neural findings were not specific to any single stimulus category but were observed when both categories were considered together, suggesting that the identified brain regions are involved in general preference estimation rather than being influenced by a particular type of stimulus.


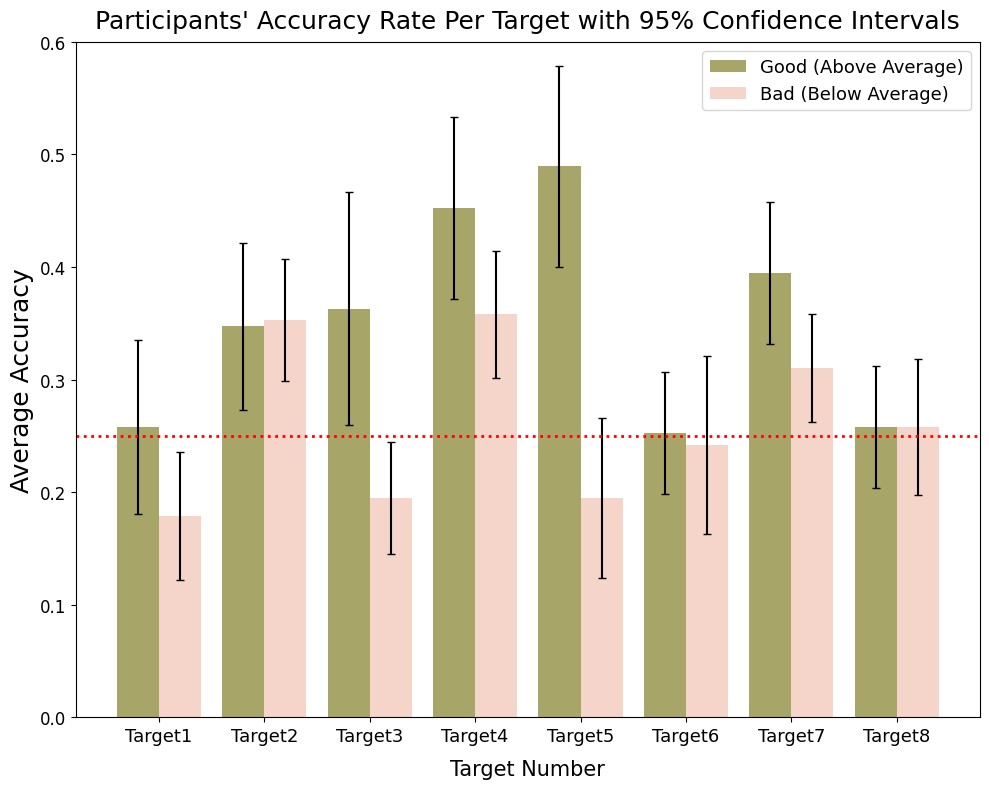


**Figure S5 | Participants’ Accuracy Rate per Target: Comparison of Good and Bad performers**

This figure illustrates the variations in participants' accuracy rates when estimating the preferences of each target, categorized into Good performers (above average; n = 19) and Bad performers (below average; n = 19), based on the group mean accuracy score (M = 0.3066). The mean (0.3066) and median (0.3067) values were nearly identical, allowing for a convenient split into two equally sized groups. Red dotted line indicates the chance performance level (0.25).

Overall, participants in the Bad performers group exhibited consistently lower accuracy rates across most targets. However, the differences in accuracy between Good and Bad performers varied in magnitude. Specifically, significant differences were observed for Targets 3, 5, and 7, where Good performers demonstrated notably higher accuracy rates compared to Bad performers. These findings underscore specific targets where low-performing participants struggled the most, reinforcing the general trend of lower accuracy among this group.

Conversely, for Targets 2, 6, and 8, the differences between Good and Bad performers were not statistically significant, suggesting that these targets did not distinctly separate performance levels. Despite these exceptions, the overall pattern remains clear, with Good performers typically achieving higher accuracy than Bad performers.

Additionally, we examined whether gender distribution influenced these findings. Since the targets were evenly divided between male (Targets 1–4) and female (Targets 5–8), and both significant differences and non-differences were observed across male and female targets alike, we conclude that the gender balance does not skew the results. This confirms that the observed performance differences are robust and reflect meaningful variations between Good and Bad performers, independent of target gender.


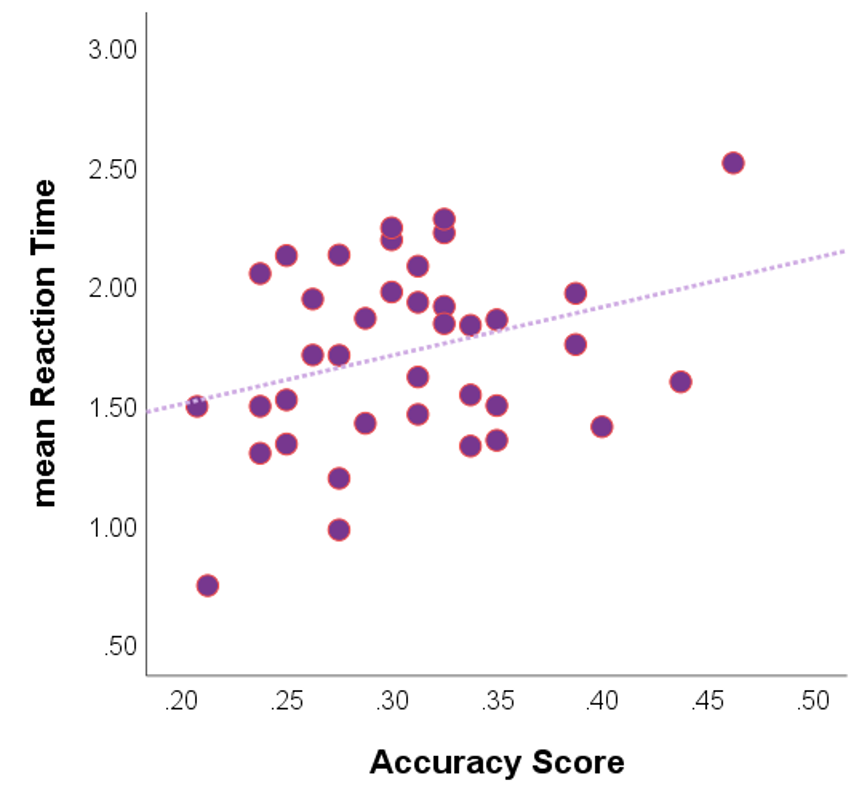


**Figure S6 | Correlation Between Mean Reaction Time (RT) and Accuracy Score**

This figure illustrates the relationship between each participant’s mean reaction time (RT) and their accuracy score. The scatter plot shows individual data points, with a fitted regression line indicating a marginally significant positive correlation (*r*(36) = 0.306, *p* = 0.062). This trend suggests that participants who achieved higher accuracy in estimating preferences tended to take longer to respond, possibly reflecting increased task engagement or more comprehensive processing of sensory information to refine their estimations.

To put this in context, our IS-RSA analysis revealed that when reaction time was included as a covariate, the involvement of the mPFC in preference estimation was no longer significant, while the insula remained significant. This suggests that the mPFC may function as a hub for integrating sensory information, and its role may be modulated by the time participants spend processing the task. By contrast, the insula's consistent involvement indicates its potentially more stable role in this process.

Together, these findings underscore the complexity of the neural mechanisms underlying preference estimation and highlight the importance of considering reaction time as a factor that can influence brain activity patterns.


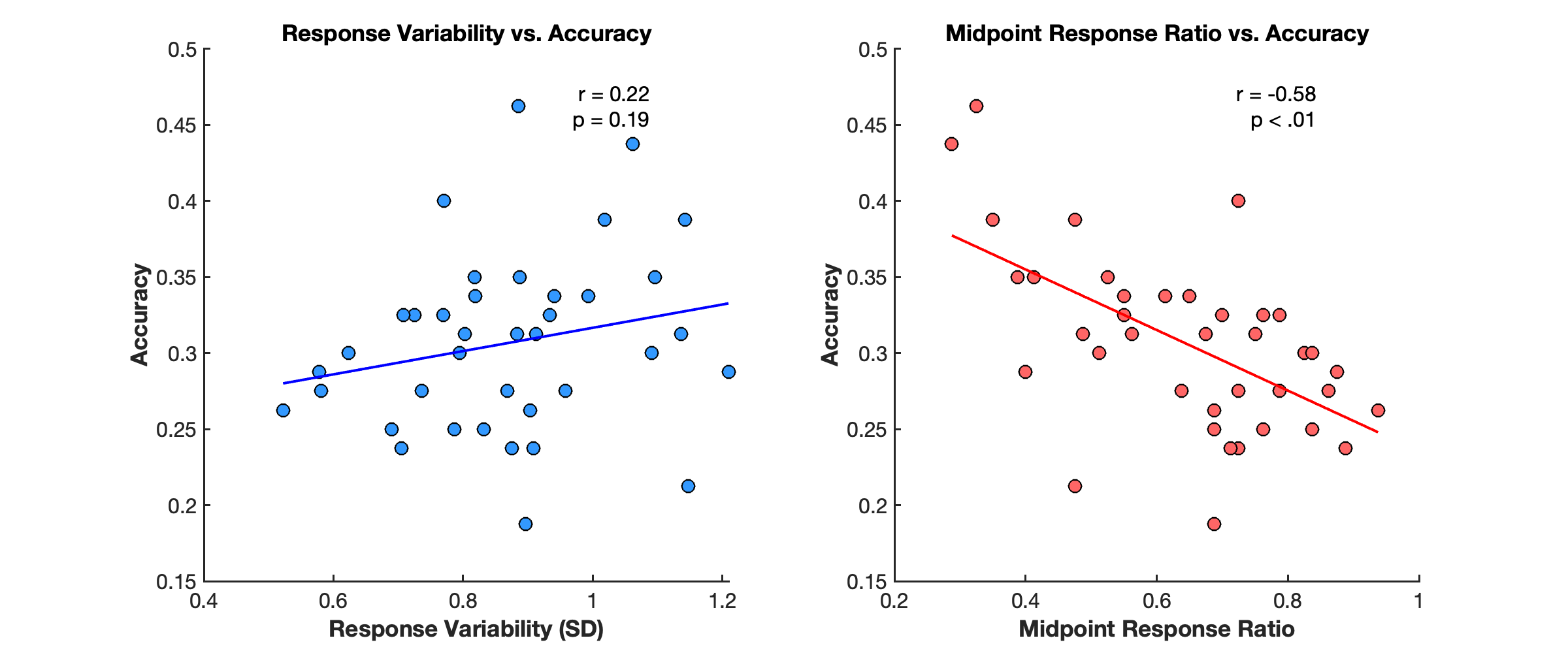


**Figure S7 | Correlation Between Response Variability & Accuracy, and Midpoint Response Ratio & Accuracy**

This figure illustrates two key analyses regarding response patterns and accuracy. The left panel presents the correlation between response variability (standard deviation of participants’ responses) and accuracy. No significant relationship was found (*r*(36) = 0.22, *p* = 0.19), indicating that greater response variability did not predict higher accuracy. The right panel presents the correlation between the proportion of midpoint responses (ratings of 2 or 3) and accuracy. A significant negative correlation was found (*r*(36) = -0.58, *p* <0.01), suggesting that participants who predominantly used midpoint responses exhibited lower accuracy.

These findings support the argument that accuracy is not merely a function of response style but rather reflects the ability to make fine-grained distinctions in preference estimation. While response variability itself did not predict accuracy, participants who avoided extreme anchors (1 or 4) tended to perform worse, suggesting that midpoint reliance may hinder precise social inference.


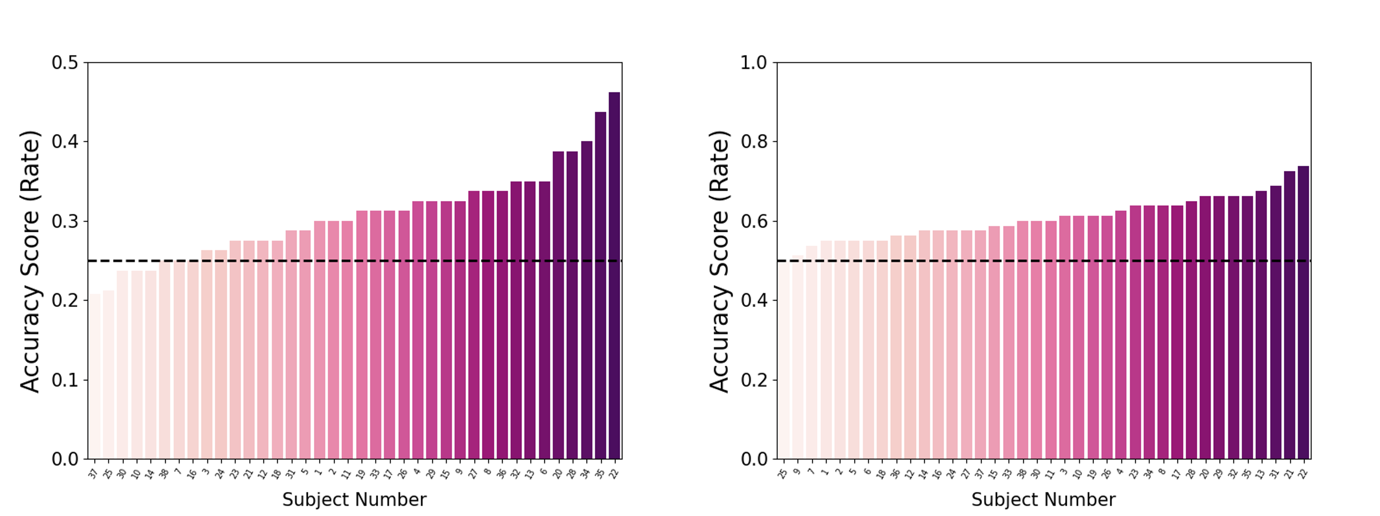


**Figure S8 | Comparison of fine-grained and categorical accuracy across participants**

This figure illustrates the accuracy scores for each participant based on two different measures. The left panel shows fine-grained (exact) accuracy, where responses were counted as correct only if they exactly matched the target’s rating. The right panel shows categorical accuracy, where ratings of ‘like’ (3) and ‘strongly like’ (4) are combined into one category, as are ratings of ‘dislike’ (2) and ‘strongly dislike’ (1). The dashed lines indicate chance performance levels (0.25 for fine-grained accuracy and 0.50 for categorical accuracy).
